# Cumulative evaluation data: pediatric airway management simulation courses for pediatric residents

**DOI:** 10.1186/s41077-017-0044-3

**Published:** 2017-08-01

**Authors:** Sawsan Alyousef, Haifa Marwa, Najd Alnojaidi, Hani Lababidi, Muhammad Salman Bashir

**Affiliations:** 1 Specialized Children Hospital, King Fahad Medical City, Riyadh, Saudi Arabia; 2 Center for Research, Education & Simulation Enhanced Training (CRESENT), King Fahad Medical City, Riyadh, Saudi Arabia; 3 Research Center, King Fahad Medical City, Riyadh, Saudi Arabia; 4 Al-Maarefa Medical University, Riyadh, Saudi Arabia

## Abstract

**Objectives:**

To utilize cumulative evaluation data of the pediatric airway management simulation-based learning course on knowledge and practical skills of residents in the Saudi Commission for Health Speciality (SCFHS) in order to measure its efficacy and areas for improvement.

**Methods:**

The evaluation is a retrospective cohort study that compares pre- and post-test (knowledge and skills) of a pediatric airway management simulation course. The 2-day course has been conducted four times annually at CRESENT and is comprised of interactive lectures on airway management and crew resource management, a demonstration of fundamentals of intubation, three skill stations, and six case scenarios with debriefing. Our evaluation data includes all pediatric residents who attended the course between January and December 2015.

**Results:**

Forty-six residents participated, of whom 30 (65.2%) are male and 16 (34.78%) are female. Overall, there is statistically significant improvement between the pre-test and post-test knowledge and practical skill scores. The pre-test scores are significantly different between the four different resident levels with *p* values of 0.003 and <0.001 respectively. However, there are no statistically significant differences in the post-test scores among the four different resident levels with *p* values of 0.372 and 0.133 respectively. The practical skill assessment covers four main domains. Improvements were noted in pharmacology (811%), equipment setup (250%), intubation steps (200%), and patient positioning (130%). The post-test scores are similar in all practical skill categories for the four different residency levels.

**Discussion:**

Our outcome-based evaluation strategy demonstrated that residents met the course learning objectives. The pediatric airway management simulation course at CRESENT is effective in improving the knowledge and practical skills of pediatric residents. Although the greatest improvement is noted among junior residents, learners from different residency levels have comparable knowledge and practical skills at the end of the course. Things that can be improved based on our study results include stressing more the type and dosages of the medications used in airway management and mandating the course for all junior pediatric residents. Although residents scored well, specific knowledge and skill elements still led us to targeted areas for course excellence. Similar courses need to be integrated in the pediatric residency curriculum. Further research is needed to study skill retention and more importantly its impact on patients’ care. Although resource-intensive, the use of cumulative evaluation data helped to focus quality improvement in our courses.

## Introduction

Airway management is a common procedure performed in the Pediatric Intensive Care Unit (PICU) and Emergency Department (ED). In contrast to the clinical experience with elective intubation in the operating room, intubation of critically ill patients has been associated with several complications [1]. Most airway management situations in the PICU/ED are emergent, leaving providers with limited time to perform a systematic airway assessment. Critically ill patients frequently have significant cardiac and pulmonary disease and limited physiologic reserve [2, 3]. These complicating factors commonly result in significant pre-oxygenation difficulty, limitations in the choice and dose of commonly used induction and paralytic agents, and less time for intubation preparation and performance. Loss of muscle tone, secretions and upper airway edema also increase the technical difficulty of glottis visualization and successful procedure performance [4, 5]. The number of intubation attempts increases the risk of adverse tracheal intubation associated events such as severe hypoxia, hypotension and cardiac arrest [2, 6, 7]. It also increases the risk of intraventricular hemorrhage in low birth weight neonates [8].

The pediatric airway management simulation course is conducted four times per year at CRESENT, King Fahad Medical City (KFMC), Riyadh, Saudi Arabia. The course was adapted from the American College of Chest Physicians (ACCP). The course has been selected by pediatric residents at KFMC in the top five most common simulation courses needed. We wanted to utilize cumulative evaluation data of the pediatric airway management simulation-based learning course on knowledge and practical skills of residents in the Saudi Commission for Health Speciality (SCFHS) in order to measure its efficacy and areas for improvement.

## Methods

We used an outcomes-based approach of evaluation to inform future courses [9–11]. We chose to use evaluation data over a one-year period since our participant numbers are quite small and we wanted to ensure weight of data and accommodate several iterations of the course. Although the course is standardized there may be variations based on participant engagement. This evaluation design is a retrospective cohort pre-test post-test that compares knowledge and practical assessments of residents attending the pediatric airway management simulation course.

### Course description

**Fig. 1:**
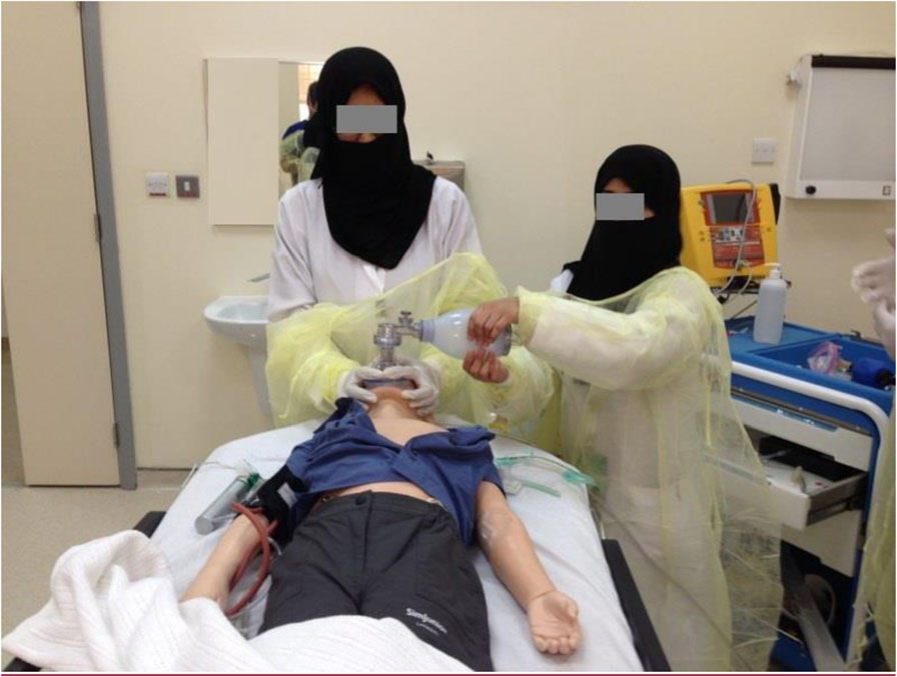
Trainees are practicing at Basic Airway Skill Station

The two-day course is conducted four times annually at CRESENT. Twelve to 18 participants per course learn with an instructor to resident ratio of 1:6. Learning objectives are listed in Table 1. During the introduction, the course director introduces the instructors and simulation technicians. The course director and instructors are all pediatric intensivists with experience in simulation-based education including targeted training on using simulation to support learning. The faculty has participated in faculty development courses at CRESENT, namely the FD-Sim course, and IMS course from the Center of Medical Simulation (CMS). The residents tour the simulation center and are familiarized with the simulation rooms, debriefing rooms, simulators and all the equipment. The course director introduces the basic assumption and safety container [12]. The simulation rooms resemble PICU rooms and equipped with SimJunior® or SimBaby®, crash cart with a defibrillator and airway tools for infants and children. The course utilizes three skills station rooms: 1) basic airway tools and infant and child intubation heads (Fig. 1), 2) advanced airway tools such as video laryngoscope and intubation bronchoscope and 3) surgical airway tools for cricothyroidotomy utilizing TraumaMan® manikin.

**Table 1:** Learning objectives of the pediatric airway management course

Domain	Learning objectives
Patient care	1. Demonstrate observation of universal precautions at all times
	2. Demonstrate clinical skills of competent performance of airway management
Medical knowledge	3. Define respiratory failure
	4. Describe the basic anatomy and physiology of the paediatric airway
Practice-based learning and improvement	5. Demonstrate management of simple and difficult airway diseases
	6. Demonstrate sound decision-making based on available medical information
Interpersonal and communication skills	7. Demonstrate the use of crew resource management
	8. Demonstrate effective interdisciplinary teamwork

The course schedule includes: 1) two interactive lectures on airway management and crew resource management, 30 min each, 2) a demonstration session on fundamentals of intubation for 60 min, 3) three skills stations, 4) six case scenarios, two on each concept of can ventilate-can intubate, can ventilate-can’t intubate and can’t ventilate-can’t intubate. Each scenario is followed by video debriefing. All activities are done in group fashion. When the scenario necessitates, moulage is performed on SimJunior® or SimBaby®.

Debriefing usually follows the 10-min case scenario and lasts for 20 min. Three phases of debriefing are used: 1) reaction discussion of both feelings and facts, 2) understanding on how to improve or sustain performance through exploring, discussion and teaching and generalization, and 3) summary and take home messages.

### Evaluation instruments

Evaluation of participants is done at the beginning of the course and then at the end. Residents complete a pre-test of 20 multiple choices questions with equal weight and a maximum score of 100%. The test was developed by one the authors (SA) to reflect the learning objectives and was face validated with a group of experts in the field. In the practical skills test, the resident is given a scenario of a patient with respiratory failure and to manage. A 31-point checklist with equal weight adapted from ACCP (Table 2) is used to assess the resident’s performance. The checklist is divided into 4 parts: equipment setup, patient positioning, pharmacology and intubation steps.

**Table 2:** Pediatric airway management procedural skills checklist

#	Equipment Setup	Yes	No
1	BVM with O2 on at 10L (positioned on left)		
2	10cm PEEP valve (positioned on left)		
3	Oral and/or nasal airway (positioned on left)		
4	Free flowing IV		
5	Suction on: coming from right, positioned on right		
6	ET tube-proper size for age with stylet (positioned on right)		
7	Cuff checked: 10cc syringe attached		
8	ETCO2 detector (positioned on left)		
9	Laryngoscope handle and blade with light on: positioned on left		
10	Difficult airway cart immediately available		
#	Patient Positioning	Yes	No
1	Headboard off		
2	Side rails down		
3	Head positioned to align airway axis		
4	Bed height appropriate		
#	Pharmacology	Yes	No
1	Midazolam 0.05-0.1 mg/kg bolus or Fentanyl 1-2 μg/kg bolus ready or ketamine 1-2mg/kg AND Succinylcholine 1-2 mg/kg bolus or Rocuronium 0.6-1.2mg/kg bolus		
2	Atropine or Epinephrine available		
#	Intubation Steps	Yes	No
1	Put on personal protective equipment (gloves, mask, eye protection minimum)		
2	Pre-oxygenation performed		
3	Verbalize airway assessment		
4	Intubator verbalizes explicit review of plan/back up with cutoffs		
5	Induction agent given		
6	Ability to ventilate considered/confirmed		
7	Cricoid pressure applied (optional)		
8	Neuromuscular agent given		
9	Intubation technique appropriate		
10	Intubator halts intubation efforts and initiates BVM if saturation decreases by 5% or <90%		
11	Airway adjunct (oral/nasal) considered/employed if sat <90%		
12	Intubation successful		
13	ETT set at appropriate length for age at gum line (3x tube size in mm)		
14	Placement confirmed with 2 indicators (positive ETCO2, breath sounds, O2 saturation)		
15	Intubator does not let go of tube until it is secured		

Table 2 displays a total of 31 points for practical skill assessment checklist which is subdivided into four main categories as equipment setup with 10 divisions, patient positioning with four divisions, pharmacology with two divisions, and lastly intubation steps with 15 divisions. Each trainee will be assessed using this checklist at pre-test and post-test, that is before and after the course

### Study population

The study includes all pediatric residents under Saudi Commission for Health Specialties (SCFHS) training program who attended the pediatric airway management simulation course between January 2015 and December 2015 at CRESENT, KFMC.

### Statistical analysis

Categorical variables of gender and level are presented as numbers and percentages. Continuous variables of age, pre-test and post-test scores are expressed as Mean ± S.D. Paired sample t-test / ANOVA is applied to determine the mean significant difference among pre-test and post-test scores. A *p*-value of less than 0.05 is considered as statistically significant. All data is entered and analyzed through statistical package SPSS version 22.

The study is approved by KFMC IRB Committee.

## Finding

A total number of 46 trainees are included from four pediatric airway management simulation courses at CRESENT in 2015, 30 residents (65.22%) are males and 16 (34.78%) are females. The breakdown of the residents by level of training is presented in Table 3. Overall, there is statistically significant improvement between the pre-test and post-test knowledge and practical skills scores (Table 4). Eighty-seven per cent of residents show improvement in their knowledge test score, 13% had no change and none had a decline in their mean scores that is statistically significance (*p*<0.001). However, all residents, who participated in these courses, showed improvement in their practical skills. A comparative analysis of pre-test and post-test mean scores of the four categories of practical skills assessment showed significant statistical improvement with *P*-value <0.001 as presented in Table 5.

**Table 3:** Residency level of the trainees

Residency level	Number	Percentage
R1	17	37%
R2	15	33%
R3	9	20%
R4	5	10%
Total	46	100%

**Table 4:** Pre-test and post-test scores for knowledge and practical skills

Item	Pre-test (%)	Post-test (%)	*p* value
Knowledge	48.0 ± 22.1	70.4 ± 15.5	<0.001
Practical skills	17.5 ± 10.9	89.4 ± 9.6	<0.001

Table 4 is comparing pre-test and post-test pediatric airway management knowledge scores which showed a significant *p* value of <0.001, also it is comparing pre-test and post-test pediatric airway management practical skills scores which showed as well significant *p* value of <0.001

**Table 5:** Comparative analysis of pre-test and post-test mean scores of the four domains of practical skills

Item		*n*	Mean ± S.D.	*p* value
Equipment setup (10 points)	Pre	46	2.15 ± 1.66	<0.001
	Post	46	7.74 ± 1.51	
Patient positioning (4 points)	Pre	46	0.78 ± 0.96	<0.001
	Post	46	3.54 ± 0.81	
Pharmacology (2 points)	Pre	46	0.15 ± 0.36	<0.001
	Post	46	1.63 ± 0.61	
Intubation steps (15 points)	Pre	46	4.02 ± 2.52	<0.001
	Post	46	12.28 ± 1.87	

Table 5 is comparing pre-test and post-test scores separately for each domain of practical skill assessment which showed significant *p* value of <0.001 for all of the domains

The breakdown of the pre-test and post-test knowledge and practical skills scores by resident level is presented in Table 6. The pre-test knowledge and practical skills scores are significantly different between the resident levels with *p*-values of 0.003 and <0.001. On the contrary, there are no statistically significant differences in the post-test knowledge and practical skills among the resident levels with *p*-values of 0.372 and 0.133.

**Table 6:** Breakdown of knowledge and practical skill scores by resident level

Resident level	Knowledge test (20 points)			Practical skills (31 points)		
	Pre	Post	*p* value	Pre	Post	*p* value
R1 (*n* = 17)	6.8 ± 2.9	16.6 ± 2.7	<0.001	4.3 ± 2.7	23.9 ± 3.6	<0.001
R2 (*n* = 15)	10.6 ± 5.8	17.7 ± 2.3	<0.001	7.2 ± 2.7	25.3 ± 3.6	<0.001
R3 (*n* = 9)	11.4 ± 4.8	18.2 ± 2.1	0.001	8.9 ± 3.9	25.8 ± 4.4	<0.001
R4 (*n* = 5)	15.2 ± 3.1	17.6 ± 2.3	0.202	13.2 ± 7.7	28.2 ± 1.7	0.003

Table 6 is comparing pre-test and post-test knowledge practical skill scores for each residency level separately which showed improvement of the scores at all residency levels, and by comparing the pre-test knowledge and practical skill scores, it showed significant difference between the four different resident levels with *p* values of 0.003 and <0.001 respectively. On the contrary, there were no significant differences in the post-test knowledge and practical skills among the four different resident levels

The practical skills assessment is divided into four domains. The best improvement between pre-test and post-test is in the pharmacology 9% vs. 82% respectively (811%), followed by equipment setup 22% vs. 77% (250%), intubation steps 27% vs. 81% (200%) and finally patient positioning 20% vs. 46% (130%). The breakdown of practical skills domains by resident level is presented in Table 7. Only 2 categories of practical skills show statistically significant differences in the pre-test among the four residents’ levels: equipment setup (*p*<0.001) and intubation steps (*p*<0.001). The post-test scores are similar in all practical skills categories for the different residency levels; equipment setup (*p*=0.168), patient positioning (*p*=0.815), pharmacology (*p*=0.093) and intubation steps (*p*=0.369).

**Table 7:** Breakdown of practical skill scores domains by resident level

Resident level	Equipment setup (10 points)			Patient setup (4 points)			Pharmacology (2 points)			Intubation steps (15 points)		
	Pre-test	Post-test	*p* value	Pre-test	Post-test	*p* value	Pre-test	Post-test	*p* value	Pre-test	Post-test	*p* value
R1 (*n* = 17)	1.3 ± 1.2	7.3 ± 1.5	<0.001	0.5 ± 0.9	3.5 ± 0.7	<0.001	0.1 ± 0.3	1.4 ± 0.8	<0.001	2.4 ± 1.5	11.8 ± 1.6	<0.001
R2 (*n* = 15)	2.3 ± 0.9	7.7 ± 1.4	<0.001	0.7 ± 0.8	3.5 ± 1.1	<0.001	0.1 ± 0.3	1.7 ± 0.5	<0.001	4.1 ± 1.9	12.4 ± 1.9	<0.001
R3 (*n* = 9)	2.1 ± 1.7	7.9 ± 1.7	<0.001	1.0 ± 1.3	3.7 ± 0.5	<0.001	0.3 ± 0.5	1.8 ± 0.4	<0.001	5.4 ± 1.9	12.4 ± 2.5	<0.001
R4 (*n* = 5)	4.6 ± 2.5	9.0 ± 1.2	0.008	1.4 ± 0.9	3.8 ± 0.5	<0.001	0.4 ± 0.6	1.7 ± 0.1	0.001	6.8 ± 4.1	13.4 ± 0.9	0.008

Table 7 displays the four main domains for the pre-test and post-test practical skill scores for each residency level separately which shows significant *p* values for all residency levels at all the domains, and by comparing pre-test scores among the four resident levels, only equipment setup and intubation steps showed significant *p* value of <0.001, while post-test scores showed almost similar scores for all the residency levels

## Discussion

This study shows the importance and effectiveness of the pediatric airway management simulation course for pediatric residents under SCFHS training programs. The striking results are the improvement in all assessed categories of practical skills ranging from 130% to 800%, which makes a strong argument to mandate such courses to all pediatric residents. Several studies have shown the efficacy of airway management training on improving intubation skills [13, 14]. However, the evidence of its impact on reducing the hazards and the risk on the patients remains limited [15].

A key element in assessing the effectiveness of simulation-based educational activity, is to document measurable improvement in knowledge, behavior and skills [16, 17]. Unlike other courses, we have not relied on resident satisfaction with the course for quality improvement, but measured their knowledge and skills before and after the course. The detailed and comprehensive outcomes-based evaluation in this course provides sufficient data for us to maintain elements of the course and improve others. Things that can be improved based on our study results include: stressing more the type and dosages of the medications used in airway management and mandating the course for all junior pediatric residents. On the other hand, by integrating the evaluation into the course schedule, it facilitates ease of data collection. It also has an orienting impact for all residents as the opening activity in the course. The practical skills assessment in particular is labor intensive; however, it is an imperative tool for accurate measurements of the course’s impact.

The course focuses on skills such as teamwork, crew resource management and communication techniques. These skills together with proper preparation of the intubation equipment, having them organized in predetermined way and the use of cognitive aid have crucial effects on the success of safe intubation [13, 18, 19]. Similar results have been reported with training of otolaryngology residents on advanced airway skills [20].

The pediatric airway management course at CRESENT targets all pediatric residents. There are clear differences in the pre-test scores among the four levels of residents which give validity to the assessment tool used. However, the junior residents show the greatest improvement in their post-test scores to the level of the seniors which strengthens the effectiveness of the course. Training for airway management including endotracheal intubation should be conducted early during residency to get the maximum benefit [21]. The importance of integrating airway management course into training programs for residents who manage critically ill children is essential as it reflects directly on patients’ outcome and safety [15, 17]. Attending advance life support courses once every two years is insufficient to improve intubation management [22]. A simulation-based education curriculum for a residency program is best constructed in a modular fashion [23]. A pediatric airway management course is one of these modules that best be administered early in the residency program.

## Conclusion

The pediatric airway management simulation course at CRESENT is effective in improving the knowledge and practical skills of pediatric residents. Although the greatest improvement is noted among junior residents, learners from different residency levels have comparable knowledge and practical skills at the end of the course. Similar courses need to be integrated in the pediatric residency curriculum preferably at early stage of residency programs. Further research is needed to study skills’ retention and more importantly its impact on patients’ care. Our outcomes-based evaluation strategy has provided targeted insight to the strengths and areas for development in the course which we have acted upon.
